# Crosstalk of the Androgen Receptor with Transcriptional Collaborators: Potential Therapeutic Targets for Castration-Resistant Prostate Cancer

**DOI:** 10.3390/cancers9030022

**Published:** 2017-02-28

**Authors:** Daisuke Obinata, Kenichi Takayama, Satoru Takahashi, Satoshi Inoue

**Affiliations:** 1 Department of Urology, Nihon University School of Medicine, Tokyo 173-8610, Japan; obinata.daisuke@nihon-u.ac.jp (D.O.); takahashi.satoru@nihon-u.ac.jp (S.T.); 2 Department of Functional Biogerontology, Tokyo Metropolitan Institute of Gerontology, Tokyo 173-0015, Japan; ktakayama-tky@umin.ac.jp; 3 Division of Gene Regulation and Signal Transduction, Research Center for Genomic Medicine, Saitama Medical University, Saitama 350-1241, Japan

**Keywords:** androgen receptor, androgen receptor signaling pathway, coregulator, octamer transcription factor 1, pyrrole-imidazole polyamide

## Abstract

Prostate cancer is the second leading cause of death from cancer among males in Western countries. It is also the most commonly diagnosed male cancer in Japan. The progression of prostate cancer is mainly influenced by androgens and the androgen receptor (AR). Androgen deprivation therapy is an established therapy for advanced prostate cancer; however, prostate cancers frequently develop resistance to low testosterone levels and progress to the fatal stage called castration-resistant prostate cancer (CRPC). Surprisingly, AR and the AR signaling pathway are still activated in most CRPC cases. To overcome this problem, abiraterone acetate and enzalutamide were introduced for the treatment of CRPC. Despite the impact of these drugs on prolonged survival, CRPC acquires further resistance to keep the AR pathway activated. Functional molecular studies have shown that some of the AR collaborative transcription factors (TFs), including octamer transcription factor (OCT1), GATA binding protein 2 (GATA2) and forkhead box A1 (FOXA1), still stimulate AR activity in the castration-resistant state. Therefore, elucidating the crosstalk between the AR and collaborative TFs on the AR pathway is critical for developing new strategies for the treatment of CRPC. Recently, many compounds targeting this pathway have been developed for treating CRPC. In this review, we summarize the AR signaling pathway in terms of AR collaborators and focus on pyrrole-imidazole (PI) polyamide as a candidate compound for the treatment of prostate cancer.

## 1. Introduction

Prostate cancer is the major cause of death from cancer among males in Western countries. For example, the American Cancer Society has estimated 180,890 new cases of prostate cancer and 26,120 deaths from the disease in the United States in 2016. The Australian Institute of Health and Welfare estimated 18,138 new diagnoses and 3398 deaths from prostate cancer in 2016. This amounts to 21.4% and 12.8% of all male deaths from cancer in each country in 2016. In Japan, although prostate cancer is the seventh-leading cause of cancer death, recently both the number of cases and the mortality rate due to prostate cancer have increased significantly. The increased population of older males is presumed to be one of the contributors in Japan.

The androgen receptor (AR) signaling pathway plays an integral role in the progression of prostate cancer. The AR is a member of the steroid hormone receptor superfamily. The AR is activated by ligands, such as dihydrotestosterone (DHT), and then functions as a transcription factor to modulate the expression of its target genes. Approximately 80%–90% of prostate cancers are androgen-dependent at the time of diagnosis [1,2,3,4,5]. Since the finding in the 1940s that castration inhibits the progression of prostate cancer [6,7], androgen deprivation therapy (ADT), or castration, has become the most effective and widely used treatment for unresectable prostate cancer, which includes metastasis and recurrence after local therapies [8,9,10,11]. Through the combination of luteinizing hormone-releasing hormone (LH-RH) analogs and anti-androgens, ADT decreases the production of androgens and inhibits androgen binding to the AR. ADT can inhibit the progression of prostate cancer for up to 3 years, however, prostate cancer cells eventually adapt to low testosterone levels and progress to castration-resistant prostate cancer (CRPC). Surprisingly, even in a low testosterone environment, AR and its target genes, including prostate-specific antigen (PSA), are still highly expressed in the majority of CRPC lesions [10,11,12]. Indeed, the rise in serum PSA levels in patients that no longer respond to ADT shows that CRPC is not hormone-insensitive. In addition, anti-androgen drugs can work as AR agonists in CRPC [13]. Some tumours acquire genomic amplifications of the AR gene, which increases their sensitivity to androgens and maintains AR signaling under the low testosterone environment of ADT [14,15]. About 30% of CRPC cases have amplifications of the AR locus [16]. Using AR-overexpressing cells, an in vitro study showed that first generation anti-androgen drugs promote AR nuclear translocation, DNA binding and co-activator recruitment [17]. AR stability also relates to AR hypersensitivity. Under physiological androgen levels, the AR is involved in a negative feedback where it suppresses the expression of genes that promote its translation. In ADT, the testosterone level is too low for the AR to inhibit these genes, but is still sufficient to stimulate AR signaling in CRPC [18]. Furthermore, deregulation of the interplay of AR with AR collaborating factors commonly occurs in CRPC cells [19].

The extragonadal androgens synthesized in adrenal or CRPC cells are one of the key mechanisms for sustaining AR signaling in CRPC. They activate the cytochrome P450 (CYP) family, which facilitates the unusual conversion of cholesterol to androgen under low testosterone conditions. Thus, the expression of androgen-dependent genes is induced by a very small amount of androgens under castration [20]. Abiraterone acetate and enzalutamide strongly target the AR pathway and improve cancer specific survival in the case with CRPC [21,22,23]. Abiraterone is a dual inhibitor of the 17α-hydroxylase and 17,20-lyase, which belong to the CYP17 family and play a key role in the novel androgen synthesis pathway in CRPC cells [24]. Enzalutamide is a novel AR antagonist that binds directly to AR with a higher affinity than bicalutamide or flutamide and targets multiple steps including AR nuclear translocation, DNA binding, and co-activator recruitment [21]. Despite the development of these notable drugs in the last decade, CRPC still evolves to acquire further resistance to these drugs. Aberrant AR function and cross-talk with factors that activate the AR pathway are assumed to be involved in this cancer evolution. Thus, the study of AR signaling pathways and their collaborative factors will facilitate greater understanding of the mechanisms underlying the progression of advanced prostate cancer as well as the development of novel drugs.

This article reviews the AR signaling pathway in CRPC as well as the development of novel therapeutic medicines targeting AR collaborators, especially collaborative DNA binding transcription factors (TFs).

## 2. AR Structure and Collaborating Factors in AR Signaling Pathway

The AR contains an N-terminal domain (NTD; 555 amino acids encoded by exon 1), a DNA-binding domain (DBD; 68 amino acids encoded by exons 2 and 3), a hinge region, and a ligand binding domain (LBD; 295 amino acids encoded by exons 4–8) [25]. The NTD includes the activation function (AF) 1 element, which enables the transactivation of the AR [26]. The LBD is located in the C-terminal region where androgens, such as DHT, bind in the first step of the androgen signaling pathway. After activation by ligands, the AR translocates into the nucleus and then binds to specific DNA sequences, called androgen response elements (AREs). The DBD plays an important role at this stage involving AR nuclear localization, homodimer formation, and specific DNA binding.

The increased frequency of functional AR mutations in CRPC enhances resistance to ADT. In addition, ADT drugs mediate a conformational change in the AR [27,28]. The proportions of AR mutations in prostate cancer are 40% in the NTD, 49% in the LBD, and 7% in the DBD [29]. Important mutations cause gain-of-function in the LBD [30], one of the most common of which is T878A. Because this mutation broadens ligand specificity, the anti-androgen flutamide, as well as other steroids, become partial agonists [31,32]. This mutation can be found in approximately one-third of CRPC [33,34], whilst the other mutations appear to be rare [35].

Previous reports have shown that constitutively active AR isoforms (splice variants: ARVs) were detected in CRPC cell lines and patient tissues [36]. These ARVs have common structural characteristics of the NTD, encoded by exons 1 and 2 or exons 1 to 3, followed by a truncated C-terminal domain (CTD) originating from introns 2 or 3. Among these ARVs, AR-V7, encoded by exons 1 to 3 with the cryptic exons, is the most abundantly detected variant in prostate cancer [37]. Lacking the LBD in the CTD, it is expected that: (1) enzalutamide is unable to bind to AR-V7; and (2) AR-V7 is activated independently, despite the low androgen levels due to abiraterone acetate. A recent report showed that positive AR-V7 expression in circulating prostate cancer cells was associated with the resistance to enzalutamide and abiraterone acetate [38].

The regulation of AR-targeted gene expression requires the recruitment of coregulators to regulatory regions of the AR protein. Coregulators promote (named coactivators), or inhibit (named corepressors) AR transactivation. Although coregulators do not need to bind DNA, they recruit general TFs associated with RNA polymerase II (Rpol II) to gene promoters [39]. The actions of AR coactivators have been well characterized for *PSA*, a classical AR-regulated gene. The AR and coactivator complex first occupies the *PSA* enhancer region and then bridges to the promoter, which allows Rpol II to track to this region [40]. Since the discovery of steroid receptor coactivator-1 (SRC-1), more than 200 nuclear receptor coregulators have been identified [39,41,42,43]. The elevated expression of SRC-1, 2 and 3 is related to poor prognosis of patients with localized prostate cancer as well as CRPC [44].

In addition to AR coregulators, TFs that collaborate with AR are also important for androgen responsive gene expression. Generally, most genes are packed and condensed into nucleosomes by being wound around the four core histones [45]. Thus, nucleosomes prevent the AR from binding to AREs. Some TFs make histone modifications to support AR binding to target regions. Wang et al. identified 90 functional AR binding regions in chromosomes 21 and 22 using high-throughput technologies [46]. Interestingly, they reported that the canonical ARE (AGAACAnnnTGTTCT) [47] existed in only 10% of these AR binding regions, whilst 68% of the AR binding regions harbored non-canonical, but functional AREs where motifs for three TFs, GATA binding protein 2 (GATA2), forkhead box A1 (FOXA1), and octamer transcription factor (OCT1), were significantly enriched [46].

GATA and FoxA family members are known to play important roles in liver and gut development in mouse embryos [48]. In vivo footprinting analysis revealed both families commonly bind to their target gene elements first in nascent liver buds and gut endoderm to induce development [48,49]. Zaret et al. [48] proposed these factors as pioneer factors, which are able to bind DNA, even in condensed chromatin, and facilitate DNA binding of other factors by opening the chromatin [50,51].

Consistent with the results of liver developmental studies, one member of the FoxA family, FOXA1, works as a pioneer factor in the AR and estrogen receptor (ER) pathways in prostate cancer and breast cancer cells [52,53,54]. Interestingly, although overexpression of FOXA1 is associated with poor prognosis in prostate cancer [55], ERα-positive breast cancer with high FOXA1 expression shows favorable sensitivity to endocrine therapy [56]. Lupien et al. [57] reported that FOXA1 is recruited into target DNA regions according to the methylation of histone H3 lysine 4 (H3K4), which differs between cell types. These data indicate that the pioneer factor FOXA1 is first recruited to a specific DNA binding region, then facilitates the recruitment of other collaborating factors, and finally induces cell type specific gene expression.

GATA family proteins are also recruited to compact chromatin [54]. GATA2 and 3 are pioneer factors for prostate cancer and breast cancer [48]. GATA2 is required for AR binding in prostate cancer cells, whereas GATA3 is necessary for ER mediated gene expression in breast cancer [46,58]. High expression of GATA2 is related to high risk of prostate cancer [59]. Recent ChIP combined with genome-wide studies have shown that GATA2 promotes the AR pathway by (1) binding to enhancer regions before androgen stimulation; (2) modifying the histone code to allow the AR easy access; and (3) establishing chromatin loop formation [60]. In addition, GATA2 cooperates with FOXA1 to perform these actions regardless of the hormone status [60]. This means that GATA2 is functionally similar to FOXA1 in the AR pathway. Like FOXA1, which induces chromatin looping for AR target gene expression in CRPC cells, GATA2 establishes the loop via the recruitment of loop formation factor mediator complex subunit 1 (MED1) [60,61,62]. These data indicate that GATA2 and FOXA1 correlate with abundant AR hypersensitivity in CRPC cells.

OCT1 acts downstream of these pioneer factors. For prostate cancer cells, GATA2 and OCT1 work in a hierarchical network as GATA2 is recruited with AR, followed by OCT1 binding to its motifs [46]. OCT1 is comprised of two DNA-binding domains that are connected to each other by a flexible linker [63]. Previous reports showed that OCT1 is weakly recruited to some AR binding regions, and OCT1 reduced *TGM2* and *C20orf77* expression by inhibiting AR activity [64,65]. These data suggest that OCT1 recruitment is limited to specific AR regulated regions where it plays an OCT1 specialized function. Interestingly, some reports indicate that OCT1 is related to the cellular stress response [66,67]. Tantin et al. [67] reported that fibroblasts deficient in OCT1 showed hypersensitivity to radiation, doxorubicin, and hydrogen peroxide and harbored elevated levels of reactive oxygen species. Kang et al. [66] showed that a large number of stress response-related genes were regulated by OCT1. These stress response genes included DNA repair genes, such as poly(ADP-ribose) polymerase 1 (*PARP1*), and metabolic genes [68]. PARP1 plays an integral role in DNA repair, in addition, a recent report showed that PARP1 was recruited to AR binding regions and promoted AR function in advanced prostate cancer [69]. These data indicate that OCT1 might correlate with drug resistance in prostate cancer by enhancement of the AR and DNA repair pathways. Consistent with these reports, we previously reported that high OCT1 expression in prostate cancer tissues is related to poor prognosis and high AR expression [70]. These data raise the hypothesis that the major downstream target genes of the OCT1 and AR complex play an important role for prostate cancer progression. Using chromatin immunoprecipitation sequencing (ChIP-Seq) and microarray techniques, we identified acyl-CoA synthetase long-chain family member 3 (*ACSL3*) [71] as the most highly expressed gene regulated by AR and OCT1 in LNCaP cells [72]. In addition, we also revealed that high *ACSL3* expression in prostate cancer tissues was associated with poor patient prognosis [72].

In addition to these primary factors, several groups have subsequently identified ETS proto-oncogene 1, transcription factor (ETS1), ERG, ETS transcription factor (ERG), CCAAT/enhancer binding proteins (C/EBPs), nuclear factor I (NFI), NK3 homeobox 1 (NKX3-1), runt related transcription factor 1 (RUNX1), and forkhead box P1 (FOXP1) as other AR collaborative TFs [65,73,74,75,76,77,78]. The roles of C/EBPs and NFI in the AR signaling pathway are still unknown. Both factors have various subtypes (e.g., C/EBPα, β, NFIA, and NFIB), and each has different effects depending on AR response genes [65,79,80].

ETS1 is a member of the ETS (v-ets erythroblastosis virus E26 oncogene) family. Massie et al. [73] reported the enrichment of ETS consensus binding motifs and non-canonical AREs in about 70% of AR binding promoter regions. ETS1 was known to activate AR, as well as multiple cancer-associated pathways, which resulted in enhanced energy metabolism, cancer cell growth and survival [81,82]. Consistent with these data, Smith et al. [83] reported that increased *ETS1* expression is related to high-grade prostate cancer and the resistance to flutamide in prostate cancer cell lines. In addition, ETS1 directly interacts with AR and stimulates *NKX3-1* expression [73,84].

The NKX family belongs to the homeodomain class of TFs, which are critical regulators of whole organ development [85]. The role of NKX3-1 in tumor progression is still controversial. Since the *NKX3-1* gene region is frequently lost in prostate cancer and this leads to increase vascular endothelial growth factor-C (*VEGF-C*) expression, *NKX3-1* is known as a tumor suppressor gene [86,87]. On the other hand, a previous study showed that *NKX3-1* is an AR response gene as well as an AR collaborating TF [75]. This study suggested that NKX3-1 forms a positive autoregulatory loop with AR and FOXA1, and mediates cancer cell survival via induction of *RAB3B*, a member of the RAS oncogene family [75].

Similar to ETS1, ERG belongs to the class I ETS family (ERG, ETS1 and 2, ETS variant: ETV1–5, ELK1, ELK3, ELK4, ETS2 repressor factor: ERF, FEV, Fli-1 proto-oncogene: FLI1 and GA binding protein transcription factor alpha subunit: GABPα) and possesses oncogenic properties, which activate the phosphoinositide 3-kinase (PI3K) pathway to promote prostate cancer progression [88,89]. On the other hand, chromosomal rearrangements between TMPRSS2 and ERG (TMPRSS2:ERG), made by AR binding to the “breakpoint ARE” in this region, occur in around 50% of prostate cancers [90,91,92,93]. Interestingly, Bowen et al. [94] recently reported that NKX3-1 bound to the region adjacent to the “break point ARE” to prevent the TMPRSS2:ERG rearrangement and its expression.

Unlike ETS1, ERG has a unique role in the AR signaling pathway. Yu et al. [76] showed that approximately 44% of AR binding sites overlap with ERG binding sites where ERG repressed AR activity. Indeed, ERG represses a number of prostate epithelium-specific genes (*PSA*, solute carrier family 45 member 3: *SLC45A3*, microseminoprotein beta: *MSMB*, and secretoglobin family 1D member 2: *SCGB1D2*). In other words, these genes are prostate epithelial differentiation markers [95]. Yu et al. [76] suggest that TMPRSS2:ERG activates a malignant regulatory switch that inhibits physiological AR signaling by induction of enhancer of zeste 2 polycomb repressive complex 2 subunit (*EZH2*). TMPRSS2:ERG expression decreases during ADT, but is reactivated in the castration resistant state [96]. EZH2, which is a member of polycomb repressive complex 2 (PRC2), mediates the trimethylation of H3K27 [97]. This means that EZH2 represses target gene expression, and facilitates cellular dedifferentiation. For example, the tumor suppressive gene, DAB2 interacting protein (*DAB2IP*) was inhibited by EZH2/PRC2 [98]. *EZH2* is also overexpressed in hormone-refractory metastatic prostate cancer, suggesting EZH2 promotes AR independent growth [97]. Furthermore, Xu et al. [99] has shown that EZH2 works not only as a methyltransferase, but also as an activator of target genes that cooperate with AR. Unlike ERG, we have reported that the AR response gene *RUNX1* functions as an AR collaborative factor to maintain AR activity. In addition, EZH2 is recruited to the *RUNX1* promoter to repress its expression [77]. The *RUNX1* expression level in clinical prostate cancer tissues is negatively associated with *EZH2* expression, and decreased *RUNX1* expression is correlated with poor prognosis [77]. These data indicate that long-term ADT and high *EZH2* expression in androgen-independent prostate cancer inhibits *RUNX1* and the negative effect of *RUNX1* on prostate cancer progression. In addition to EZH2, Ma et al. [100] showed that the TMPRSS2:ERG activates SRY-box 9 (SOX9), which stimulates WNT signaling and tumor progression in a subset of prostate cancer.

Interestingly, previous reports have shown that high dose testosterone supplementation of castrate-resistant cells inhibits their proliferation [101,102]. This negative feedback mechanism of the AR signaling pathway might maintain prostate cancer in a well differentiated type of adenocarcinoma.

## 3. The Unique Features of Transcription Factors in Castration-Resistant Prostate Cancer

AR binding regions might keep changing with prostate cancer progression under a low testosterone environment. Recently, Sharma et al. [103] elucidated the differences in AR binding regions between ADT naïve prostate cancer and CRPC. Notably, 44% of genes with AR binding sites unique to CRPC showed no response to androgen in prostate cancer cell lines [103]. These AR binding sites are enriched in promoter regions and predominantly included E2F transcription factor (E2F), v-myc avian myelocytomatosis viral oncogene homolog (*MYC*), and signal transducer and activator of transcription (STAT) motifs compared to those in ADT naïve and prostate cancer cell lines [103].

E2F-1 activates genes related to G_1_–S transition and DNA synthesis and induces cell cycle progression [104]. The expression of *E2F-1* is regulated by the tumor suppressor gene RB transcriptional corepressor 1 (*RB1*). RB1 inhibits G_1_–S transition related gene expression by directly obstructing the transactivation domain of E2F and the promoter activity of these genes [105]. Since *RB1* loss is frequently observed in CRPC, the RB1/E2F-1 complex could play a significant role in tumor progression. A previous report suggested that loss of *RB1* enhances AR activity via *E2F-1* activation to induce resistance to ADT [106].

c-MYC is known as an oncogenic transcription factor that regulates ribosomal RNA expression, glutamine metabolism, and energy and reactive oxygen species [107,108,109]. Bernard et al. [110] reported that c-MYC was regulated by the AR and was required for AR-dependent and AR-independent growth in AR positive prostate cancer cell lines. Previous fluorescence in situ hybridization data showed the specific amplification of the *c-MYC* gene in 72% of CRPC [111,112]. Some c-MYC repressed genes, *Bin1* and *MXI1*, were inactivated in advanced prostate cancer [113,114]. Consistent with the report by Yu et al. [76] about the TMPRSS2:ERG/EZH pathway, Sun et al. [115] also reported that TMPRSS2:ERG activates *c-MYC* and represses prostate epithelial differentiation genes.

STAT3 is regulated by the Janus kinase (Jak) family/interleukin 6 (IL-6) and is also oncogenic, promoting cytosolic dimerization, nuclear translocation and DNA binding [116,117,118]. STAT3 activation is observed in 82% of prostate cancer tissues compared to matched adjacent non-cancer tissues, and elevated STAT3 activity was correlated with a malignant phenotype [119]. Interestingly, Culig et al. [120] reported that IL-6 activates AR in androgen depleted conditions to promote the growth of almost all prostate cancer cell lines. However, IL-6 stimulation inhibited LNCaP cell proliferation regardless of STAT3 activation. In addition, a recent report showed that inhibition of IL-6/STAT3 signaling in a phosphatase and tensin homolog (PTEN)-deficient prostate cancer model promotes cancer progression [121]. These data indicate that the effect of STAT3 on prostate cancer progression is still controversial. Reinforcing the report by Sharma et al. [103], a recent study shows that the pluripotency transcription factor Nanog homeobox (NANOG) alters FOXA1 and AR target genes during reprogramming of androgen-dependent prostate cancer cells to CRPC [122].

Collectively, these studies suggest that the role of the AR signaling pathway in prostate cancer progression is more complicated than expected, because AR collaborating TFs are entangled with each other and have differing effects on AR activity depending on testosterone levels and the duration of anti-androgen drug treatment.

## 4. Development of Novel Drugs

### 4.1. Pyrrole-Imidazole Polyamide

**Figure 1:**
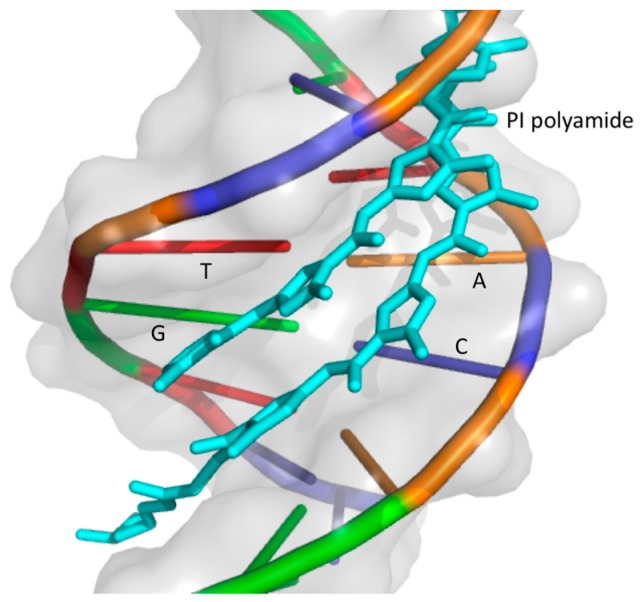
A schematic view of pyrrole-imidazole (PI) polyamide binding to a target DNA sequence. Image of 1CVY [124] created with Open-Source PyMOL Molecular Graphics System, Version 1.7, Schrödinger, LLC.

Different classes of drugs are under investigation to inhibit AR collaborative TFs. In this section, we review the development of one new class of compounds, pyrrole-imidazole (PI) polyamides, before discussing specific examples of compounds that target AR collaborative TFs in the following section. PI polyamides are small synthetic molecules made up of *N*-methylimidazole (Im) and *N*-methylpyrrole (Py) amino acids, the side by side pairings of which recognize and attach to the minor groove of DNA with high affinity and sequence specificity [123,124,125]. Im/Py pairs recognise G/C nucleotides and Py/Py pairs bind to A/T and T/A nucleotides (Figure 1) [126,127].

In addition, the C-terminal β-alanine residue next to dimethylpropylamine (Dp) and the γ-aminobutyric acid turns a unit, which enforces an antiparallel hairpin configuration and enhances both DNA binding affinity and specificity [124,128,129]. Vector-assisted delivery systems are not necessary for PI polyamide translocation to the nucleus. Following PI polyamide binding to DNA, the minor groove is widened and the major groove is bent and compressed to block TFs binding [130]. Unlike most DNA targeted therapies, PI polyamides bind to DNA non-covalently without a drug delivery system [131]. In addition, PI polyamides are fully resistant to biological degradation by nucleases and do not induce unnecessary normal cell damage and carcinogenesis [132]. These are advantages of PI polyamides compared to other chemical drugs.

The pharmacokinetics of PI polyamides provide promise for future clinical applications. Previous reports have shown that PI polyamides are not absorbed from the intestine [133]. After transvenous distribution in rat organs, PI polyamides were excreted into urine and bile without any metabolism [133,134]. Matsuda et al. showed that PI polyamides accumulated in nuclei of kidney cells in rats and were maintained for about two weeks without any drug delivery system [135,136]. Recently, Igarashi et al. [137] studied the possible clinical applications of PI polyamides using a primate model. They developed an ointment including a PI polyamide targeting human transforming growth factor beta (TGF-β) 1 and tested for hypertrophic scars in marmosets. The PI polyamide bound to keratinocyte nuclei in marmosets and suppressed hypertrophic scarring without any side effects [137]. These reports are fundamental evidence for the clinical application of PI polyamides and increasing interest in their use for AR and some AR collaborative TFs, such as OCT1 and ETS family genes.

### 4.2. Novel Drugs Targeting TFs Related to the AR Pathway

#### 4.2.1. The Pioneer Factors (FOXA1 and GATA2)

Targeting the pioneer factor FOXA1 showed contradictory results for AR activity and prostate cancer prognosis [138]. Increasing FOXA1 activity causes indiscriminate opening of closed chromatin, attracting the AR to ARE half sites at the expense of genes with canonical ARE that promote prostate cancer progression. Conversely, inhibition of FOXA1 reprogrammed the arrangement of the AR and led to overexpression some androgen-responsive genes to promote CRPC cell growth [139]. We also reported that the AR/FOXA1 response gene *FOXP1* acts as a negative AR collaborative transcriptional factor, and represses tumor activity by binding to adjacent regions to AREs [78,140]. Interestingly, the EZH2 methyltransferase inhibitor, GSK126, promotes *FOXA1* expression and inhibits breast cancer growth via cooperation with *BRCA1* [141]. Recently, Zhao et al. [142] elucidated the dichotomous functions of FOXA1 in the AR signaling pathway. They indicated that FOXA1 reprograms the AR and GATA2 cistromes as a pioneer factor [142]. Whilst FOXA1 represses AR binding to DNA, GATA2 positively collaborates with the AR in androgen-mediated gene expression in prostate cancer [142]. Previous reports showed that GATA2 specific inhibition using the low-molecular-weight compound K-7174 [143] suppressed AR expression and the proliferation of CRPC cells [144]. Although it is not known whether this compound is suitable for clinical applications, it is ingestible and possesses beneficial effects for haematological diseases [145,146,147].

#### 4.2.2. OCT1

Whilst many studies have focused on FOXA1 and GATA2, OCT1 is often overlooked, so we have developed a novel drug targeting Oct1/AR using PI polyamides. A previous report showed that a PI polyamide targeting AREs suppressed androgen-responsive gene expression in LNCaP cells [148]. This sophisticated report showed that targeting canonical AREs was clearly effective; however, it is possible that PI polyamides that also cover non-canonical AREs might block the proliferation of CRPC even further. We identified the ACSL3 enhancer region, where AR and OCT1 regulate transcriptional activity, and developed a PI polyamide targeting OCT1 binding elements in this region [72]. This PI polyamide suppressed ACSL3 expression and CRPC cell growth. In addition, it specifically repressed global OCT1 chromatin association and AR signaling in prostate cancer cells [72]. These data reinforce the evidence that OCT1 is also important for AR recruitment to mediate global AR-response gene expression. Our study supports a novel therapeutic strategy using PI polyamides in patients with CRPC.

#### 4.2.3. ETS Family Genes

There is one report of an ETS-1 inhibitor using double-strand oligodeoxynucleotides (ODNs) that represses gastric cancer cell proliferation [149]. ODNs mimic transcription factor binding sites and act as decoys that compete with the original DNA binding sites in promoter regions [150]. Unlike PI polyamides, ODNs require improvements to the drug delivery systems to target cells and greater in vivo stability before they are suitable for clinical applications.

Since ERG was shown to be an oncogenic protein, ERG target drugs became attractive agents for prostate cancer. PARP inhibitors, a direct ERG binding small molecule (YK-4-279), a DNA-binding inhibitor targeting ETS consensus sites (DB1255), and a drug that enhances ERG ubiquitination (WP1130) are all promising compounds for prostate cancer [151,152,153,154]. In terms of TMPRSS2:ERG, we previously developed a PI polyamide targeting a common sequence in AR-related DNA break points among *TMPRSS2* and *ERG* gene loci to repress TMPRSS2:ERG expression and prostate cancer cell growth [155]. Furthermore, a recent report showed that targeting AREs downregulated TMPRSS2:ERG expression in VCaP cells and inhibited the growth of VCaP cells in vivo [156].

#### 4.2.4. NKX3-1

Ren et al. [157] developed NKX3-1 targeting compounds using RNA activation (RNAa). RNAa is system that uses small double-stranded RNA (dsRNA) that target selected gene promoter regions [158]. Transfecting the synthesized dsRNA into human cell lines causes induction of target gene expression. Ren et al. showed that increased *NKX3-1* expression by RNAa formulated in lipid nanoparticles significantly inhibited prostate tumor growth both in vitro and in vivo [157].

#### 4.2.5. C/EBP Family

Although the role of the C/EBP family in prostate cancer is still unknown, a recent report showed that RNAa targeting *C/EBPα* repressed the proliferation of pancreatic ductal adenocarcinoma cells [159]. In addition, a phase I clinical study of RNAa targeting *C/EBPα* is underway for severe liver cancer (NCT02716012).

#### 4.2.6. E2F-1

Several studies of E2F-1 inhibitors have been reported. Kaseb et al. [160] studied the efficacy of a herbal product, thymoquinone, extracted from *Nigella sativa* seeds for prostate cancer. Interestingly, thymoquinone inhibited the tumor growth of CRPC xenografts and repressed E2F-1 and AR expression [160]. Xie et al. [161] also developed a peptide binding to the *E2F-1* consensus sequence. Treatment of mice with this peptide encapsulated in PEGylated liposomes inhibited the growth of an AR negative prostate cancer cell line without toxicity [162].

#### 4.2.7. c-MYC

Like ERG, there are several agents targeting c-MYC [163,164,165,166]. Recently, Rebello et al. [167] reported the efficacy of a combination of RNA polymerase I (Rpol I) and proto-oncogene serine/threonine-protein (PIM) kinase inhibitors (CX-5461 and CX-6258) for MYC-driven prostate cancer. They showed that c-MYC is related to both Rpol I and PIM kinase activation, which were significantly inhibited by both drugs in Hi-MYC mice [167].

#### 4.2.8. STAT3

Leong et al. [168] showed that inhibiting *STAT3* using ODNs repressed head and neck cancer cell growth. In addition, Hedvat et al. showed favorable results in prostate cancer for a STAT3 inhibitor, AZD1480, which is a potent ATP competitive inhibitor of Jak2 kinase [169]. However, Fizazi et al. [170] reported an anti-IL-6 monoclonal antibody, siltuximab, inhibited *STAT3* expression, but did not find a survival improvement in patients with advanced prostate cancer.

The information about AR collaborative TFs and related drugs discussed in this section is summarized in Table 1.

## 5. Conclusions

AR collaborators, such as collaborative TFs, are important in the extraordinary hypersensitivity of the AR in CRPC. In addition, activation of AR-regulated genes promotes prostate cancer progression. Over the last decade, sophisticated technologies for investigating transcriptional networks have broadened our understanding of AR signaling in prostate cancer. Various functional studies, including our own work, have elucidated the complicated influence that AR collaborators have on prostate cancer progression. These reports provide fundamental evidence to support the premise that developing novel drugs against AR collaborators could provide promising strategies to treat CRPC. Thus, further studies of these novel candidate compounds with pre-clinical drug screening models will be crucial for developing new strategies to treat CRPC [24,171,172,173,174].

## Figures and Tables

**Table 1:** AR collaborative TFs.

Factor	Functions for AR	Efficacy for Cancer Progression	FOXA1 Interaction	Related Drugs	Reference
FOXA1	Pioneer factor	Controversial		GSK126	[141]
GATA2	Pioneer factor/Activator	Promote	+	K-7174	[143,145,146,147]
OCT1	Activator	Promote	+	PI polyamide	[72]
ETS1	Activator	Promote	−	ODNs	[149,150]
ERG	Repressor	Promote	−	PI polyamide/YK-4-279/DB1255/WP1130	[151,153,154,155]
NKX3-1	Activator	Controversial	+	RNAa	[157]
C/EBPs	Repressor	Unknown	–	RNAa	[159]
NFI	Diverse effects on gene regulation	Unknown	+	-	
RUNX1	Activator	Inhibit	−	-	
FOXP1	Repressor	Inhibit	+	-	
E2F	Activator (CRPC)	Promote	−	Thymoquinone/Peptide	[160,161,162]
MYC	Controversial (CRPC)	Promote	−	CX5461/CX6258	[167]
STAT3	Activator (CRPC)	Controversial	−	ODNs/AZD1480/Siltuximab	[168,169,170]

FOXA1: forkhead box A1; GATA2: GATA binding protein 2; OCT1: octamer transcription factor; ETS1: ETS proto-oncogene 1, transcription factor; ERG: ETS transcription factor; NKX3-1: NK3 homeobox 1; C/EBPs: CCAAT/enhancer binding proteins: NFI: nuclear factor I; RUNX1: runt related transcription factor 1; FOXP1: forkhead box P1; E2F: E2F transcription factor; MYC: v-myc avian myelocytomatosis viral oncogene homolog; STAT3: signal transducer and activator of transcription; CRPC: castration resistant prostate cancer; ODN: oligodeoxynucleotides; PI: pyrrole-imidazole; RNAa: RNA activation.
